# Supplementary figures and images for: Assessing dengue forecasting methods: a comparative study of statistical models and machine learning techniques in Rio de Janeiro, Brazil
Source: Trop Med Health. 2025 Apr 10;53:52. doi: 10.1186/s41182-025-00723-7 (PMC11984044; doi:10.1186/s41182-025-00723-7)

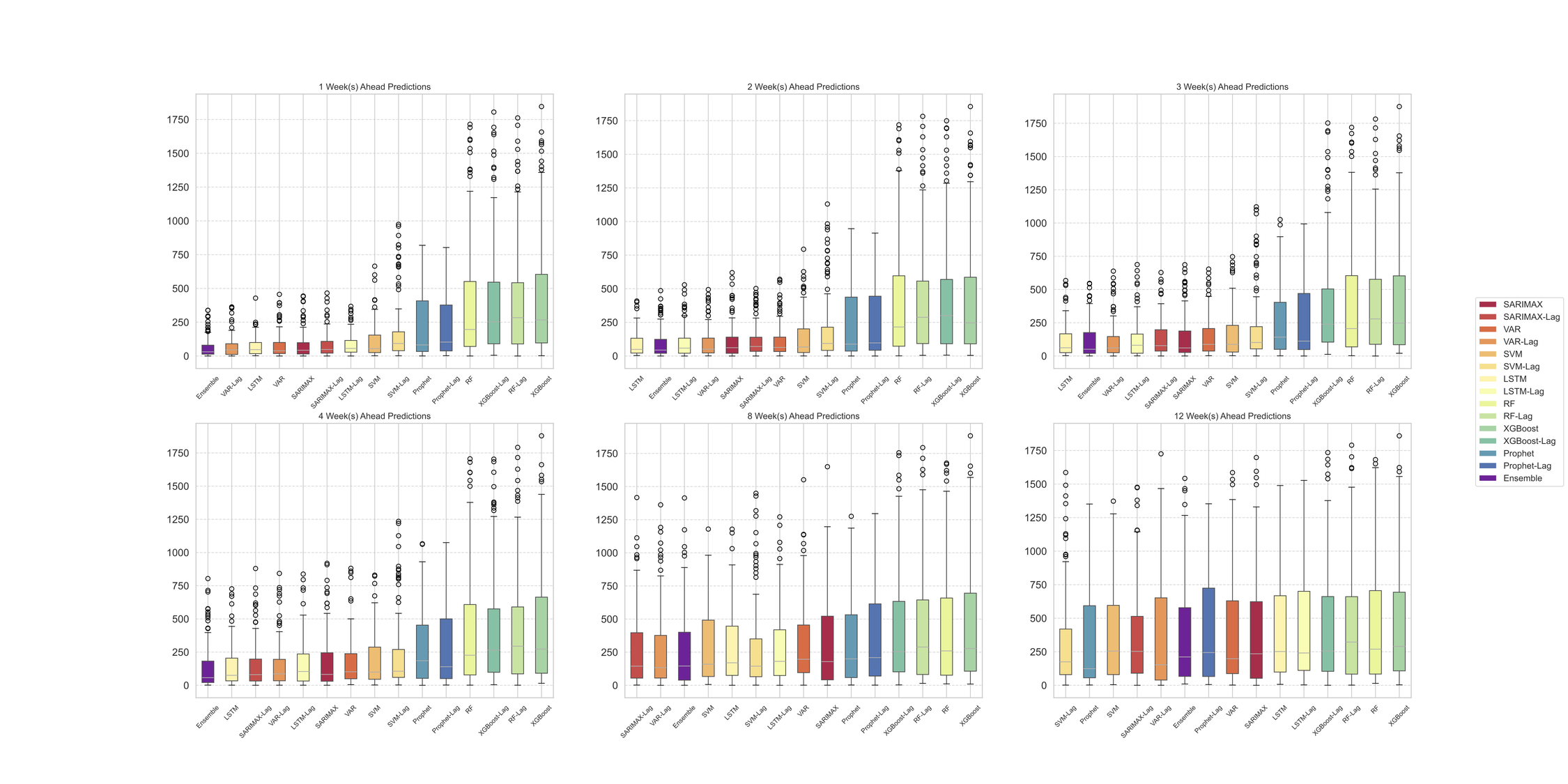

Supplement: Supplementary file 1 — Supplementary material 1. S1 Comparison of the predictions obtained by the best performing statistical and machine learning techniques for different time horizons. S2 Boxplots of the absolute errors (cases) obtained by the forecasting methods when using only cases. S3 Boxplots of the absolute percentage errors (%) obtained by the forecasting methods when using only cases. S4 Boxplots of the absolute errors (cases) obtained by the forecasting methods when including covariates. Ensemble refers to the best ensemble approach using covariates which is LSTM and SARIMAX. S5 Boxplots of the absolute percentage errors (%) obtained by the forecasting methods including covariates. Ensemble refers to the best ensemble approach using covariates which is LSTM and SARIMAX. S6 Real cases, predictions, and 95% uncertainty intervals computed with SARIMAX across various forecast horizons. S7 Real cases, predictions, and 95% uncertainty intervals computed with LSTM including covariates across various forecast horizons. S8 Computational time of each forecasting method. [file 41182_2025_723_MOESM1_ESM.zip › Supporting information/S4_Fig.tif]

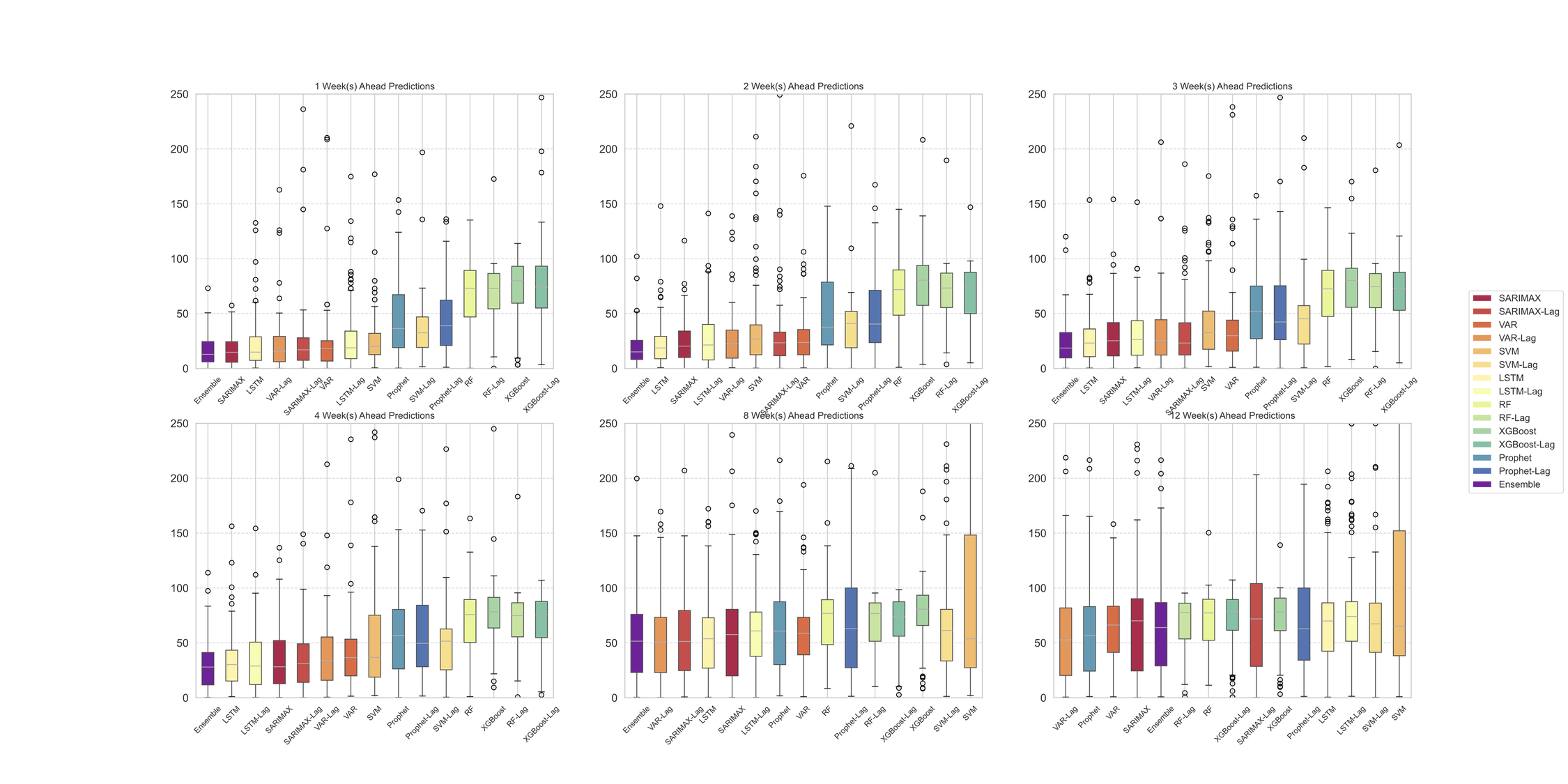

Supplement: Supplementary file 1 — Supplementary material 1. S1 Comparison of the predictions obtained by the best performing statistical and machine learning techniques for different time horizons. S2 Boxplots of the absolute errors (cases) obtained by the forecasting methods when using only cases. S3 Boxplots of the absolute percentage errors (%) obtained by the forecasting methods when using only cases. S4 Boxplots of the absolute errors (cases) obtained by the forecasting methods when including covariates. Ensemble refers to the best ensemble approach using covariates which is LSTM and SARIMAX. S5 Boxplots of the absolute percentage errors (%) obtained by the forecasting methods including covariates. Ensemble refers to the best ensemble approach using covariates which is LSTM and SARIMAX. S6 Real cases, predictions, and 95% uncertainty intervals computed with SARIMAX across various forecast horizons. S7 Real cases, predictions, and 95% uncertainty intervals computed with LSTM including covariates across various forecast horizons. S8 Computational time of each forecasting method. [file 41182_2025_723_MOESM1_ESM.zip › Supporting information/S5_Fig.tif]

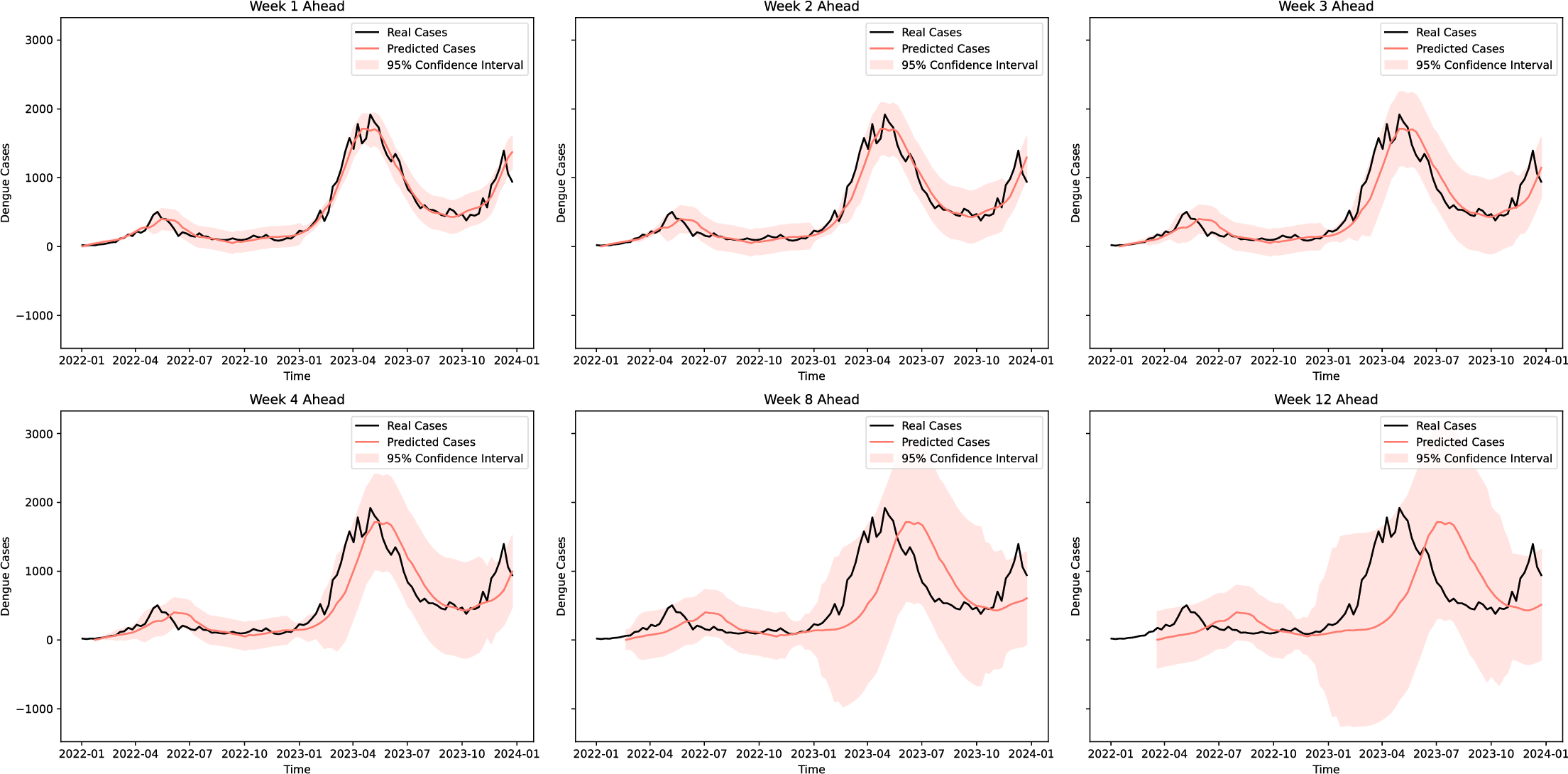

Supplement: Supplementary file 1 — Supplementary material 1. S1 Comparison of the predictions obtained by the best performing statistical and machine learning techniques for different time horizons. S2 Boxplots of the absolute errors (cases) obtained by the forecasting methods when using only cases. S3 Boxplots of the absolute percentage errors (%) obtained by the forecasting methods when using only cases. S4 Boxplots of the absolute errors (cases) obtained by the forecasting methods when including covariates. Ensemble refers to the best ensemble approach using covariates which is LSTM and SARIMAX. S5 Boxplots of the absolute percentage errors (%) obtained by the forecasting methods including covariates. Ensemble refers to the best ensemble approach using covariates which is LSTM and SARIMAX. S6 Real cases, predictions, and 95% uncertainty intervals computed with SARIMAX across various forecast horizons. S7 Real cases, predictions, and 95% uncertainty intervals computed with LSTM including covariates across various forecast horizons. S8 Computational time of each forecasting method. [file 41182_2025_723_MOESM1_ESM.zip › Supporting information/S7_Fig.tif]

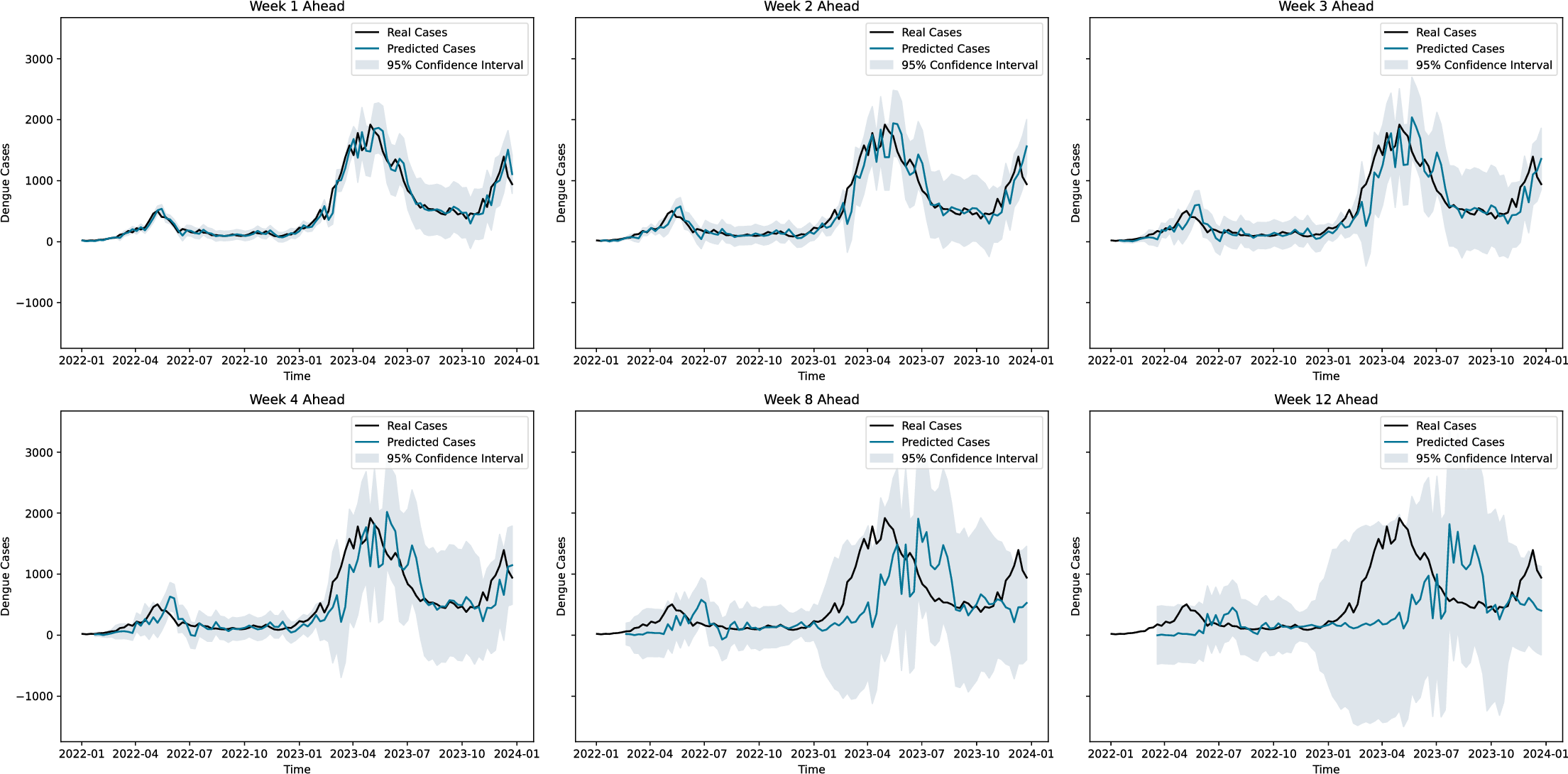

Supplement: Supplementary file 1 — Supplementary material 1. S1 Comparison of the predictions obtained by the best performing statistical and machine learning techniques for different time horizons. S2 Boxplots of the absolute errors (cases) obtained by the forecasting methods when using only cases. S3 Boxplots of the absolute percentage errors (%) obtained by the forecasting methods when using only cases. S4 Boxplots of the absolute errors (cases) obtained by the forecasting methods when including covariates. Ensemble refers to the best ensemble approach using covariates which is LSTM and SARIMAX. S5 Boxplots of the absolute percentage errors (%) obtained by the forecasting methods including covariates. Ensemble refers to the best ensemble approach using covariates which is LSTM and SARIMAX. S6 Real cases, predictions, and 95% uncertainty intervals computed with SARIMAX across various forecast horizons. S7 Real cases, predictions, and 95% uncertainty intervals computed with LSTM including covariates across various forecast horizons. S8 Computational time of each forecasting method. [file 41182_2025_723_MOESM1_ESM.zip › Supporting information/S6_Fig.tif]

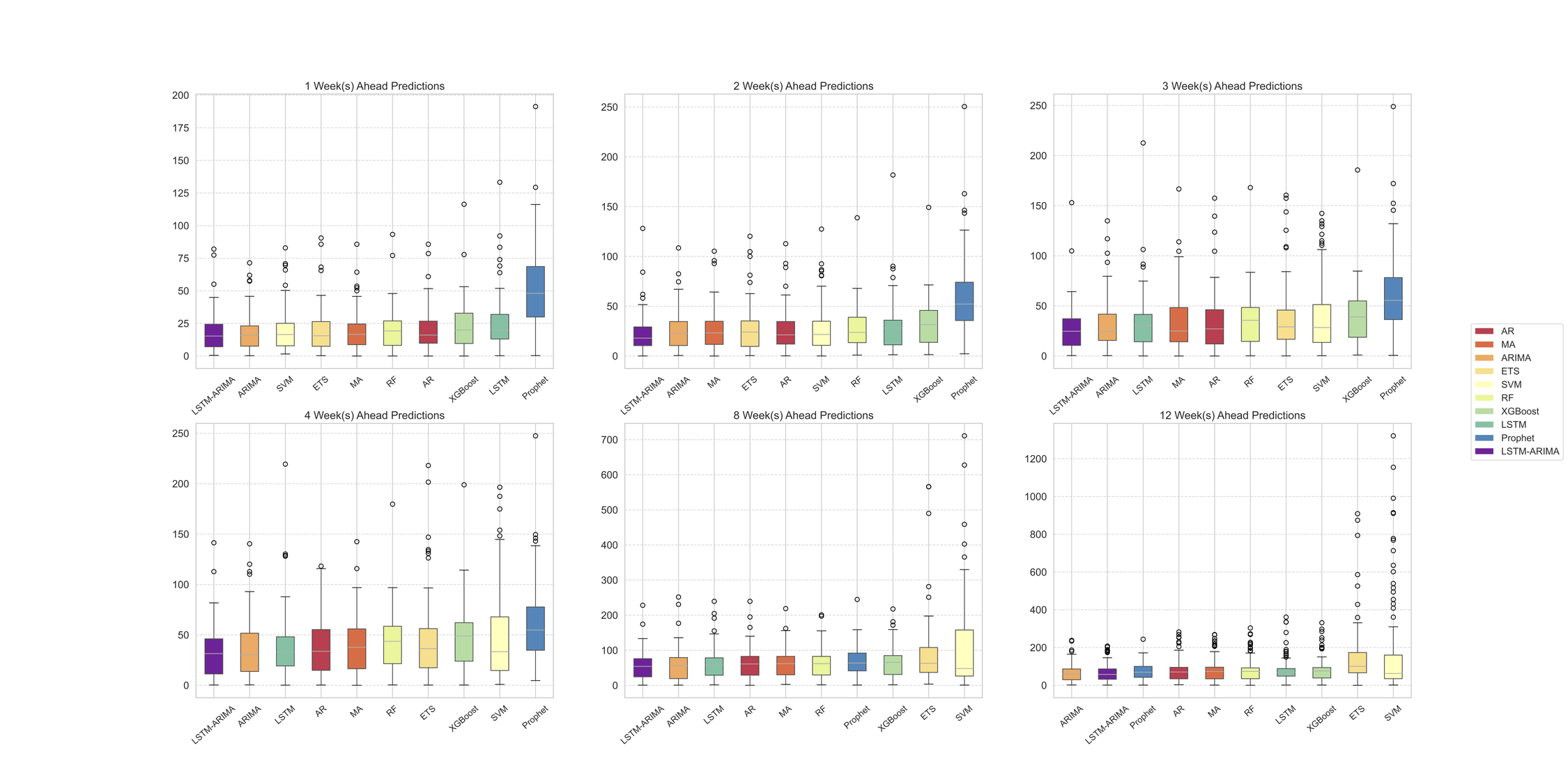

Supplement: Supplementary file 1 — Supplementary material 1. S1 Comparison of the predictions obtained by the best performing statistical and machine learning techniques for different time horizons. S2 Boxplots of the absolute errors (cases) obtained by the forecasting methods when using only cases. S3 Boxplots of the absolute percentage errors (%) obtained by the forecasting methods when using only cases. S4 Boxplots of the absolute errors (cases) obtained by the forecasting methods when including covariates. Ensemble refers to the best ensemble approach using covariates which is LSTM and SARIMAX. S5 Boxplots of the absolute percentage errors (%) obtained by the forecasting methods including covariates. Ensemble refers to the best ensemble approach using covariates which is LSTM and SARIMAX. S6 Real cases, predictions, and 95% uncertainty intervals computed with SARIMAX across various forecast horizons. S7 Real cases, predictions, and 95% uncertainty intervals computed with LSTM including covariates across various forecast horizons. S8 Computational time of each forecasting method. [file 41182_2025_723_MOESM1_ESM.zip › Supporting information/S3_Fig.tif]

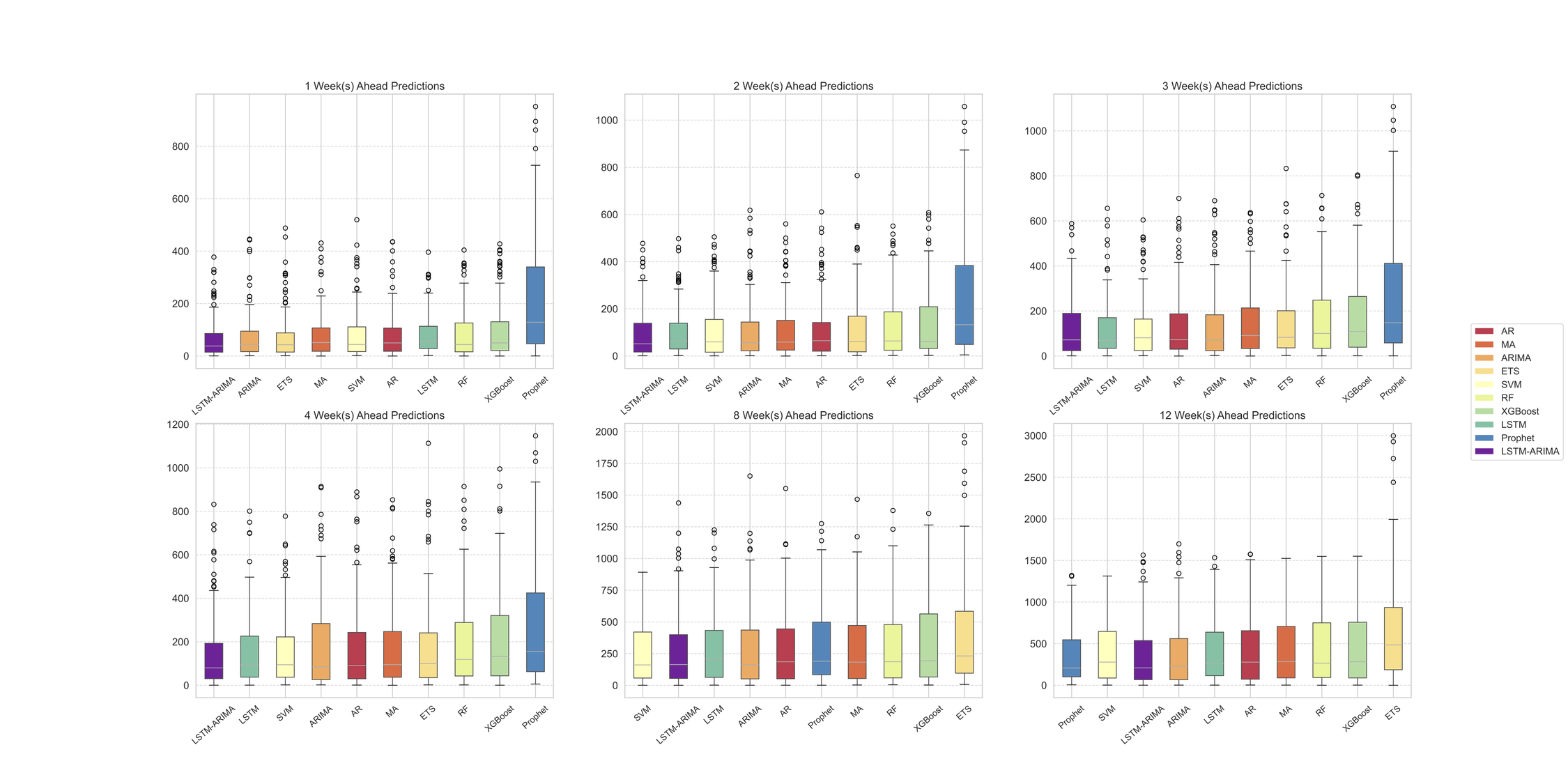

Supplement: Supplementary file 1 — Supplementary material 1. S1 Comparison of the predictions obtained by the best performing statistical and machine learning techniques for different time horizons. S2 Boxplots of the absolute errors (cases) obtained by the forecasting methods when using only cases. S3 Boxplots of the absolute percentage errors (%) obtained by the forecasting methods when using only cases. S4 Boxplots of the absolute errors (cases) obtained by the forecasting methods when including covariates. Ensemble refers to the best ensemble approach using covariates which is LSTM and SARIMAX. S5 Boxplots of the absolute percentage errors (%) obtained by the forecasting methods including covariates. Ensemble refers to the best ensemble approach using covariates which is LSTM and SARIMAX. S6 Real cases, predictions, and 95% uncertainty intervals computed with SARIMAX across various forecast horizons. S7 Real cases, predictions, and 95% uncertainty intervals computed with LSTM including covariates across various forecast horizons. S8 Computational time of each forecasting method. [file 41182_2025_723_MOESM1_ESM.zip › Supporting information/S2_Fig.tif]

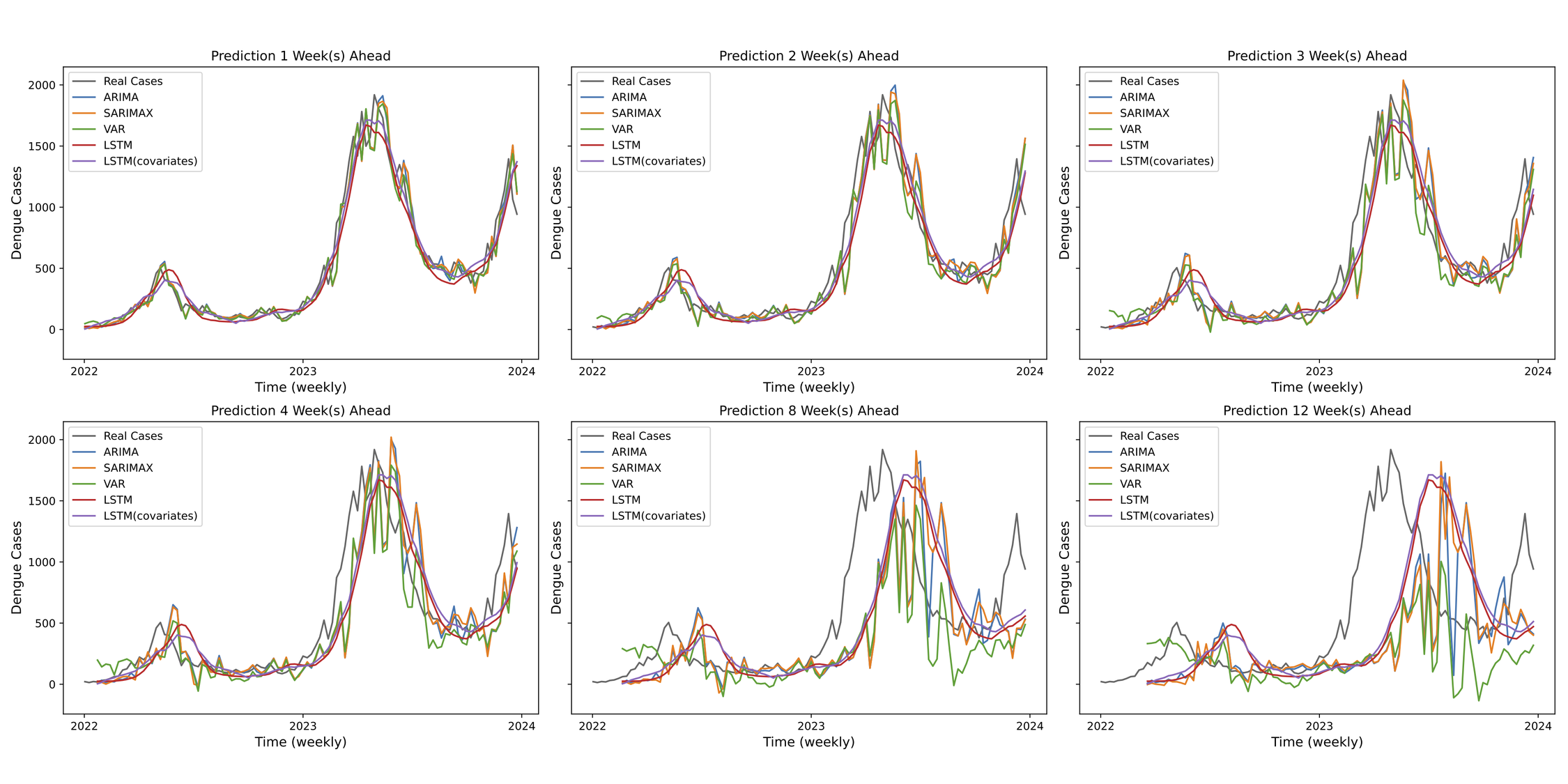

Supplement: Supplementary file 1 — Supplementary material 1. S1 Comparison of the predictions obtained by the best performing statistical and machine learning techniques for different time horizons. S2 Boxplots of the absolute errors (cases) obtained by the forecasting methods when using only cases. S3 Boxplots of the absolute percentage errors (%) obtained by the forecasting methods when using only cases. S4 Boxplots of the absolute errors (cases) obtained by the forecasting methods when including covariates. Ensemble refers to the best ensemble approach using covariates which is LSTM and SARIMAX. S5 Boxplots of the absolute percentage errors (%) obtained by the forecasting methods including covariates. Ensemble refers to the best ensemble approach using covariates which is LSTM and SARIMAX. S6 Real cases, predictions, and 95% uncertainty intervals computed with SARIMAX across various forecast horizons. S7 Real cases, predictions, and 95% uncertainty intervals computed with LSTM including covariates across various forecast horizons. S8 Computational time of each forecasting method. [file 41182_2025_723_MOESM1_ESM.zip › Supporting information/S1_Fig.tif]

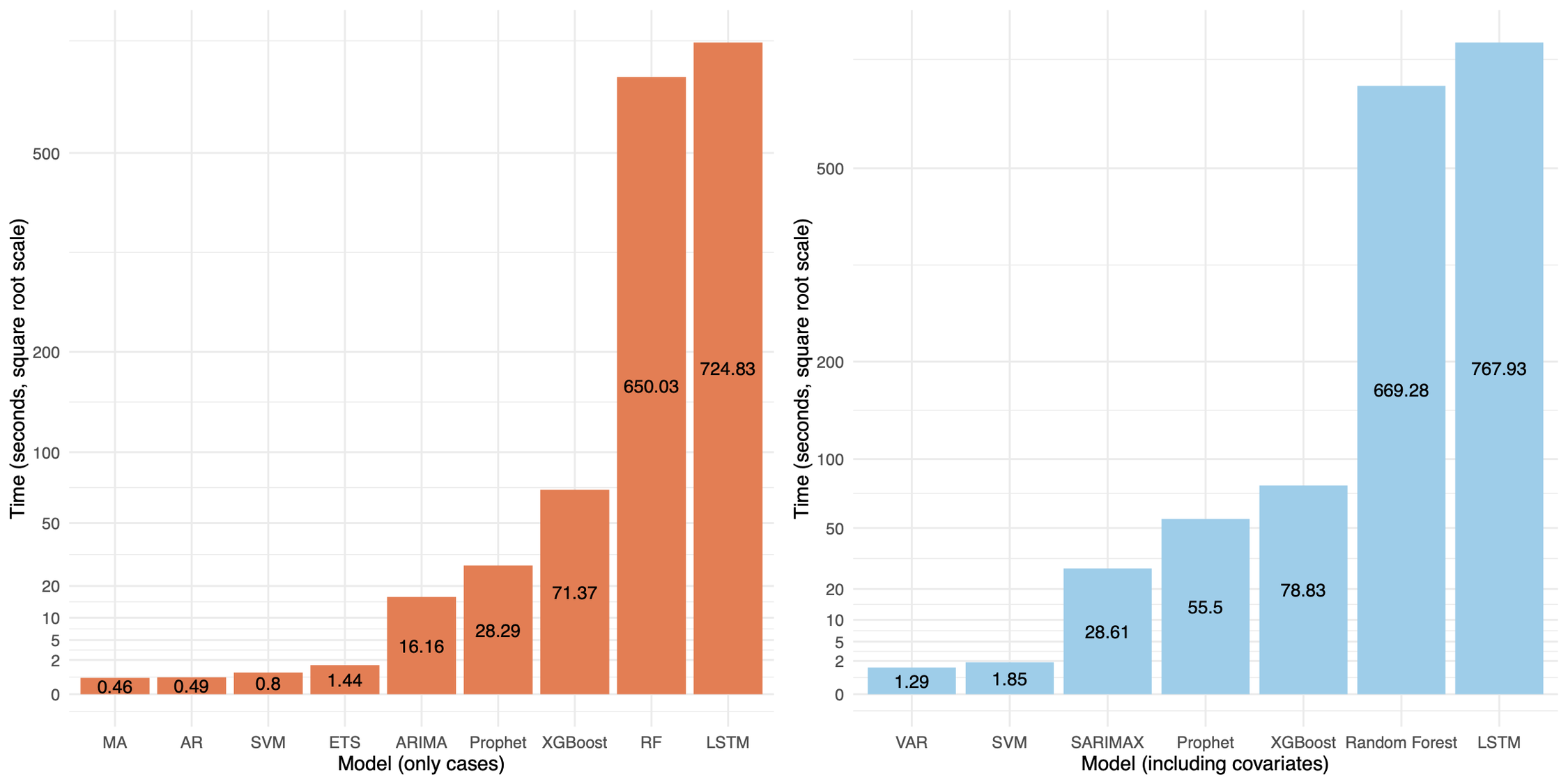

Supplement: Supplementary file 1 — Supplementary material 1. S1 Comparison of the predictions obtained by the best performing statistical and machine learning techniques for different time horizons. S2 Boxplots of the absolute errors (cases) obtained by the forecasting methods when using only cases. S3 Boxplots of the absolute percentage errors (%) obtained by the forecasting methods when using only cases. S4 Boxplots of the absolute errors (cases) obtained by the forecasting methods when including covariates. Ensemble refers to the best ensemble approach using covariates which is LSTM and SARIMAX. S5 Boxplots of the absolute percentage errors (%) obtained by the forecasting methods including covariates. Ensemble refers to the best ensemble approach using covariates which is LSTM and SARIMAX. S6 Real cases, predictions, and 95% uncertainty intervals computed with SARIMAX across various forecast horizons. S7 Real cases, predictions, and 95% uncertainty intervals computed with LSTM including covariates across various forecast horizons. S8 Computational time of each forecasting method. [file 41182_2025_723_MOESM1_ESM.zip › Supporting information/S8_Fig.tif]
